# Specific Oral Manifestations in Adults with Crohn’s Disease

**DOI:** 10.3390/jcm13133955

**Published:** 2024-07-05

**Authors:** Yavuz Cagir, Muhammed Bahaddin Durak, Cem Simsek, Ilhami Yuksel

**Affiliations:** 1 Department of Gastroenterology, Ankara Bilkent City Hospital, Bilkent, Ankara 06800, Turkey; yukselilhami@hotmail.com; 2 Department of Gastroenterology, Faculty of Medicine, Hacettepe University, Ankara 06230, Turkey; doctormbd@gmail.com (M.B.D.); cemgsimsek@gmail.com (C.S.); 3 Department of Gastroenterology, Faculty of Medicine, Ankara Yildirim Beyazit University, Ankara 06800, Turkey

**Keywords:** Crohn’s disease, extraintestinal manifestations, oral manifestations, granulomatous cheilitis, cobblestoning

## Abstract

**Background**: Oral manifestations of Crohn’s disease (CD) include non-specific lesions and specific lesions directly related to intestinal inflammation. Oral lesions that can be overlooked in CD are sometimes challenging to treat. **Methods**: In this retrospective single-center study, patients with CD aged over 18 years who complied with follow-up and treatment were included. Clinical definitions of specific oral lesions included pyostomatitis vegetans, glossitis with fissuring, lip swelling with fissuring, cobblestoning, and orofacial granulomatosis. Experienced dentists confirmed the specific lesions in each case. Three groups of patients were identified: those without oral lesions, those with non-specific oral lesions, and those with specific oral lesions. The groups were compared based on demographics, disease extent and behavior (based on the Montreal classification), extraintestinal involvement, biologic and steroid treatment, and the requirement of resective surgery. **Results:** A total of 96 patients (14.2%) with oral lesions were found among the 676 patients with CD (59.7% male, median age 38 years) who were followed for 6.83 years (IQR 0.5–29.87 years). Eight patients (1.2%, 9 lesions) had specific oral lesions, while eighty-eight patients (13%) had non-specific lesions. Orofacial granulomatosis (*n* = 3), cobblestoning (*n* = 2), glossitis with fissuring (*n* = 2), and lip swelling with fissuring (*n* = 2) were among the specific lesions. The majority of patients (75%) with specific lesions were male, and their median age was 46.5 years (range: 23–68 years). Disease localization was commonly ileocolonic (50%), and perianal disease was present in 25% of patients. Three patients were active smokers. Extraintestinal manifestations were peripheral arthritis/arthralgia (*n* = 7) and sacroiliitis (*n* = 1). All specific lesions were associated with moderate-to-severe disease. Five patients improved with biologic therapy, and two patients with immunomodulatory therapy. **Conclusions**: Specific oral lesions in CD were associated with active disease and improved with immunomodulators or biologic therapy. Close cooperation between gastroenterologists and dentists is essential for early diagnosis and optimal management of CD.

**Main Points:**

- Oral lesions that may be neglected by gastroenterologists may affect quality of life due to pain and weight loss.
- Specific oral lesions were detected in almost 1% of patients with CD.
- Peripheral arthralgia and peripheral arthritis were significantly more likely to occur among patients with oral lesions.
- A multidisciplinary approach can prevent delays in diagnosis.
- Biologic treatments are effective in specific oral lesions in CD.

## 1. Introduction

Transmural inflammation, which can affect any part of the gastrointestinal tract, from the oral cavity to the anus, is a characteristic of Crohn’s disease (CD) [1]. In addition to intestinal involvement, CD can manifest in various other organs, referred to as extraintestinal manifestations (EIMs) [2]. Well-defined EIMs in CD include arthritis (peripheral or axial), erythema nodosum, pyoderma gangrenosum, uveitis, episcleritis, primary sclerosing cholangitis, and aphthous stomatitis. However, several rare EIMs, such as polyarteritis nodosa, cutaneous vasculitis, pseudotumor cerebri, myasthenia gravis, and specific oral lesions (cobblestoning, lip swelling, orofacial granulomatosis, pyostomatitis vegetans), can be easily overlooked [3,4].

EIMs in CD might not be clinically obvious or easy to detect, posing a challenge for treating clinicians. Multidisciplinary management in CD practices can improve clinical outcomes and quality of life [5]. EIMs may present concurrently with flare-ups in the underlying CD and respond to the treatment of intestinal inflammation, or they can manifest independently of the disease course. Genetic risk factors associated with EIMs are common to both ulcerative colitis and CD [6]. Compared to nonsmokers, smokers have a higher likelihood of presenting with EIMs in CD [7].

Oral involvement in CD includes not only aphthous stomatitis but also periodontitis. Periodontitis, a chronic inflammatory disease, is characterized by gingival pain, redness, and oozing, which eventually leads to tooth loss due to damage to the alveolar bone and connective tissue. Aphthous stomatitis presents with typical aphthous lesions, similar to those found in the ileum or colon, manifesting as round or oval painful ulcers with a yellow pseudomembranous base and erythematous borders, frequently located on the buccal or labial mucosa. Rare oral manifestations of CD include orofacial granulomatosis (also known as cheilitis granulomatosis), cobblestoning, lip swelling, and glossitis with fissuring [8].

Estimates of the prevalence of oral lesions in CD vary widely, ranging from 5% to 50%. Aphthous stomatitis was shown to be the most prevalent manifestation in previous studies that reported a high frequency of oral involvement; though, the significant variance in this frequency is not well understood. However, sufficient data on specific oral lesions are still lacking, and the correlation between oral involvement and clinical outcomes has not been established [9,10,11]. This study aimed to determine the frequency and characteristics of oral lesions (specific and nonspecific), and the effects on the disease course in patients with CD.

## 2. Materials and Methods

### 2.1. Study Population and Data Collection

Patients with CD who were over 18 years of age and complied with their follow-up and treatment between June 2013 and February 2023 were included in this retrospective single-tertiary-center study. Patients were excluded from the study if they had malignant lesions, oral candidiasis, Behcet disease, or any other illness that manifested as oral lesions. The study excluded patients with indeterminate colitis, who were non-adherent to therapy, or had irregular follow-ups. While typical oral lesions were evaluated by gastroenterologists, atypical lesions were also assessed by a dentist. In each case, expert dentists confirmed the presence of specific lesions. The patients were divided into three groups: patients with no oral lesions, those with nonspecific oral lesions, and those with specific oral lesions. Demographic data, location and behavior of the disease, extraintestinal involvement, need for steroid and biologic treatment, and need for resective surgery were compared across all groups. The location and behavior of the disease was based on the Montreal classification [12]. All methodologies were conducted following the ethical guidelines outlined by the institutional research committee, the 1964 Helsinki Declaration, its subsequent amendments, or similar ethical standards. The institutional review board approved this study. (Approval date: 15 February 2023, number: E2-23-3385).

### 2.2. Management

All patients underwent a detailed medical history and physical examination, including an oral examination at the time of diagnosis and subsequent visits. Oral lesions, other extraintestinal manifestations, perianal fistula, joint examination, and skin findings were evaluated. Data such as smoking habits, family history of IBD, and surgical history were noted. Comprehensive evaluation was integrated into the civilian medical record system. Relevant data were obtained from the civil medical registry system, hospital medical registry system, and national medical system.

When only aphthous stomatitis was detected, the treatment of the intestinal disease remained unchanged, and antiseptic mouthwash and local steroid therapy were applied for symptomatic relief. In the presence of specific oral lesions, antiseptic mouthwash and local steroid treatments were also used. For patients who did not achieve clinical improvement, the clinician escalated the current treatment, regardless of the presence of active intestinal disease. While monotherapy or combination therapy with immunomodulators (IMs) was prescribed to biologically naïve patients, dose escalation or switching was managed in biologically experienced patients. Patients with nonspecific oral lesions and those without oral lesions had follow-up every three months, whereas patients with specific oral lesions were followed up in the first and third months following the initiation of treatment and then at 3-month intervals.

### 2.3. Definitions

Clinical definitions of specific oral lesions were pyostomatitis vegetans, lip/cheek swelling with fissuring, cobblestoning, and orofacial granulomatosis. Aphthous stomatitis manifests as painful, oval, or round-shaped ulcers with an erythematous edge and a yellow pseudomembranous base, resembling typical aphthous lesions observed in the colon or ileum. Orofacial granulomatosis, also known as cheilitis granulomatosis, commonly presents with chronic diffuse swelling of the lips or lower part of the face, oral ulceration, hyperplastic gingivitis, and mucosal tags resulting from granulomatous inflammation of unknown etiology [13]. Cobblestoning results from a combination of deep, transverse, and longitudinal ulcerations that divide sections of intact mucosa. These lesions typically consist of mucosal-colored papules that form firm plaques on the buccal mucosa and palate. Oral cobblestoning is considered pathognomonic for Crohn’s disease [14]. Deep linear ulcerations and lip swelling with vertical fissures commonly occur in the buccal sulci with hyperplastic folds and may also be observed in the midline lip [15]. Pyostomatitis vegetans, rarely associated with Crohn’s disease, is thought to be primarily linked to the diagnosis of ulcerative colitis. It is characterized by the development of numerous converging erythematous, white, or yellow pustules [16,17].

### 2.4. Statistical Analysis

Data analysis was performed using IBM SPSS Statistics for Windows, version 25.0 (IBM Corp., Armonk, NY, USA). The normality of the distribution of continuous variables was assessed using the Kolmogorov–Smirnov test. As all continuous variables were non-normally distributed, they were presented as median (minimum-maximum) and compared using the Kruskal–Wallis test followed by Tamhane’s T2 post hoc test. Categorical variables were expressed as frequencies (percentages) and compared using the Chi-square test. A *p* value < 0.05 was considered statistically significant.

## 3. Results

A total of 676 patients with CD, 404 (59.7%) males and 272 (40.3%) females, were included. The median age at diagnosis was 38 years with a median follow-up of 6.83 years (IQR 0.5–29.87 years). Among the study population, 255 (37.7%) were current smokers, 168 (24.9%) were ex-smokers, and 94 (13.9%) had a family history of IBD. Disease behavior was classified as inflammatory (B1) in 529 (78.3%) patients, stricturing (B2) in 45 (6.7%), and penetrating (B3) in 102 (15.1%). Perianal involvement was observed in 193 (28.6%) patients. EIMs were detected in 388 (57.3%) patients at the time of diagnosis or subsequent visits. The median Crohn’s Disease Activity Index (CDAI) score at diagnosis was 305.5 (214.5–395). In total, 366 patients (54.2%) received immunomodulators (IMs) (304 azathioprine and 62 methotrexate), while 308 patients (45.5%) underwent biologic therapy. Resective surgery was performed in 229 (33.9%) patients during the follow-up period (Table 1).

Patients were categorized into three groups: those with no oral lesions (*n* = 580), nonspecific oral lesions (*n* = 88), and specific oral lesions (*n* = 8). No significant differences were observed among the groups regarding age at Crohn’s onset, total disease duration, disease location, behavior, and perianal involvement. When comparing EIMs, peripheral arthralgia and peripheral arthritis were significantly more common in patients with specific and nonspecific oral lesions compared to those with no oral lesions (*p* < 0.001). Sacroiliitis was more prevalent in patients with specific oral lesions than in those with no oral lesions (*p* < 0.001). The need for steroid and biologic therapy and resective surgery did not differ significantly among the groups (Table 2).

Oral lesions were present in 96 (14.2%) patients. Specific oral lesions were found in eight patients (1.2%), with nine lesions in total, as one patient had both orofacial granulomatosis and lip swelling with fissuring. Nonspecific lesions were observed in 88 (13%) patients. The specific lesions included orofacial granulomatosis (*n* = 3), cobblestoning (*n* = 2), glossitis with fissuring (*n* = 2), and lip swelling with fissuring (*n* = 2). Patients with specific lesions were predominantly male (75%) with a median age of 46.5 years (range 23–68 years). All specific lesions were detected after CD diagnosis. In patients with specific oral lesions, disease location was ileal (25%), colonic (25%), and ileocolonic (50%), with perianal disease being present in 25% of patients. Disease behavior was inflammatory in seven patients and penetrating in one patient. Concurrent EIMs included peripheral arthritis/arthralgia (*n* = 7) and sacroiliitis (*n* = 1) (Table 3). All specific lesions were associated with moderate-to-severe disease activity (median C-reactive protein level 35.5 mg/L, and median CDAI 313).

**Figure 1:**
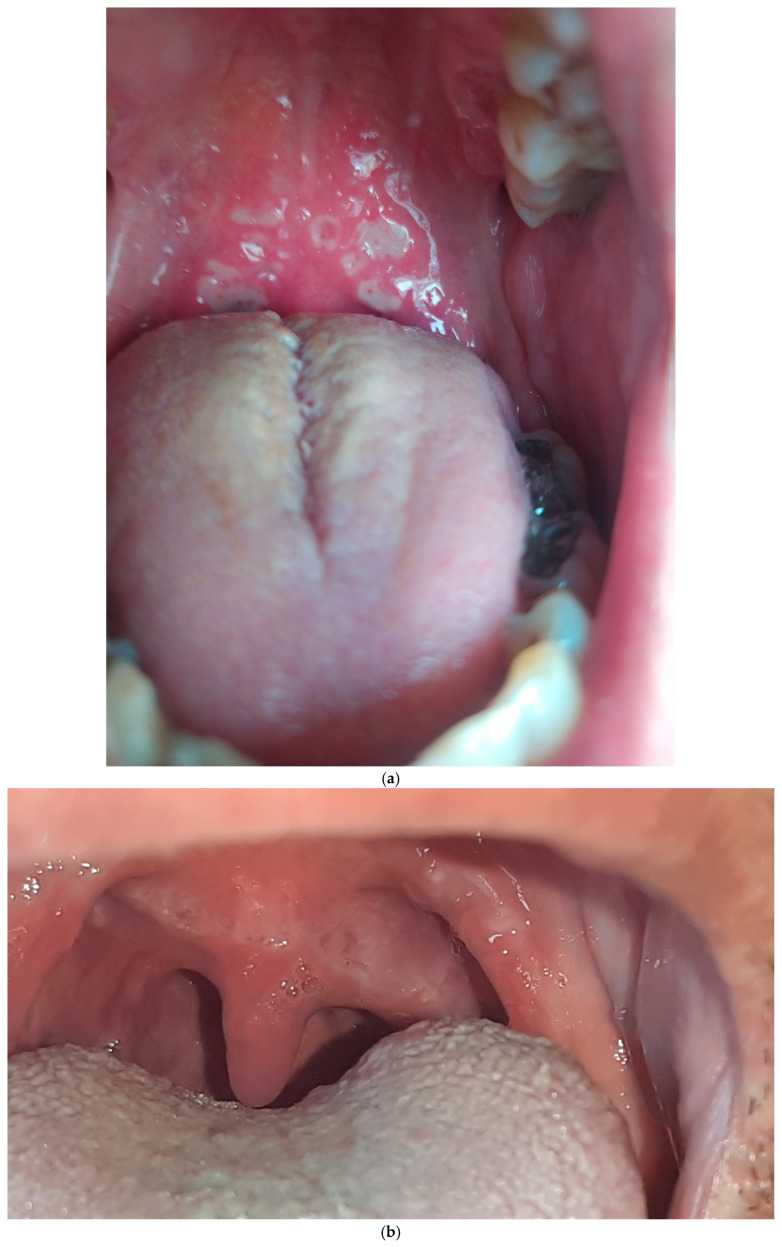
(**a**). Cobblestoning on the palatal mucosa in a patient with Crohn’s disease before treatment. (**b**). Resolution of cobblestoning on the palatal mucosa following treatment with ustekinumab.

**Figure 2:**
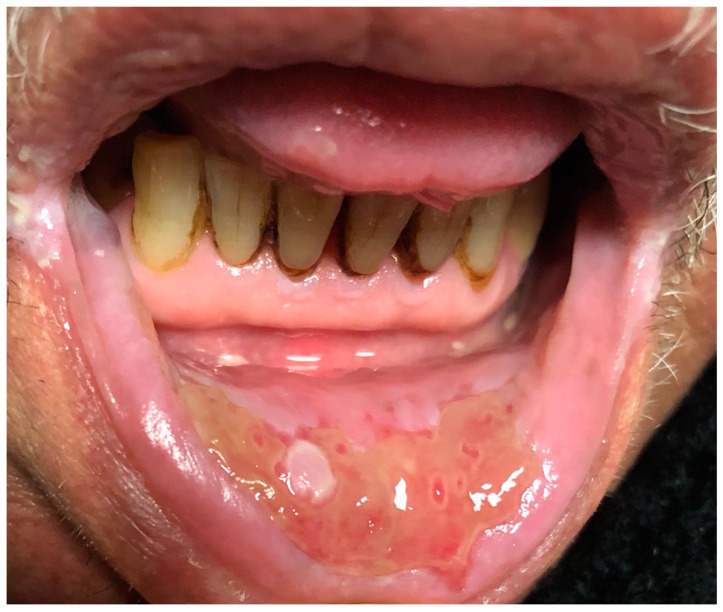
Orofacial granulomatosis.

**Figure 3:**
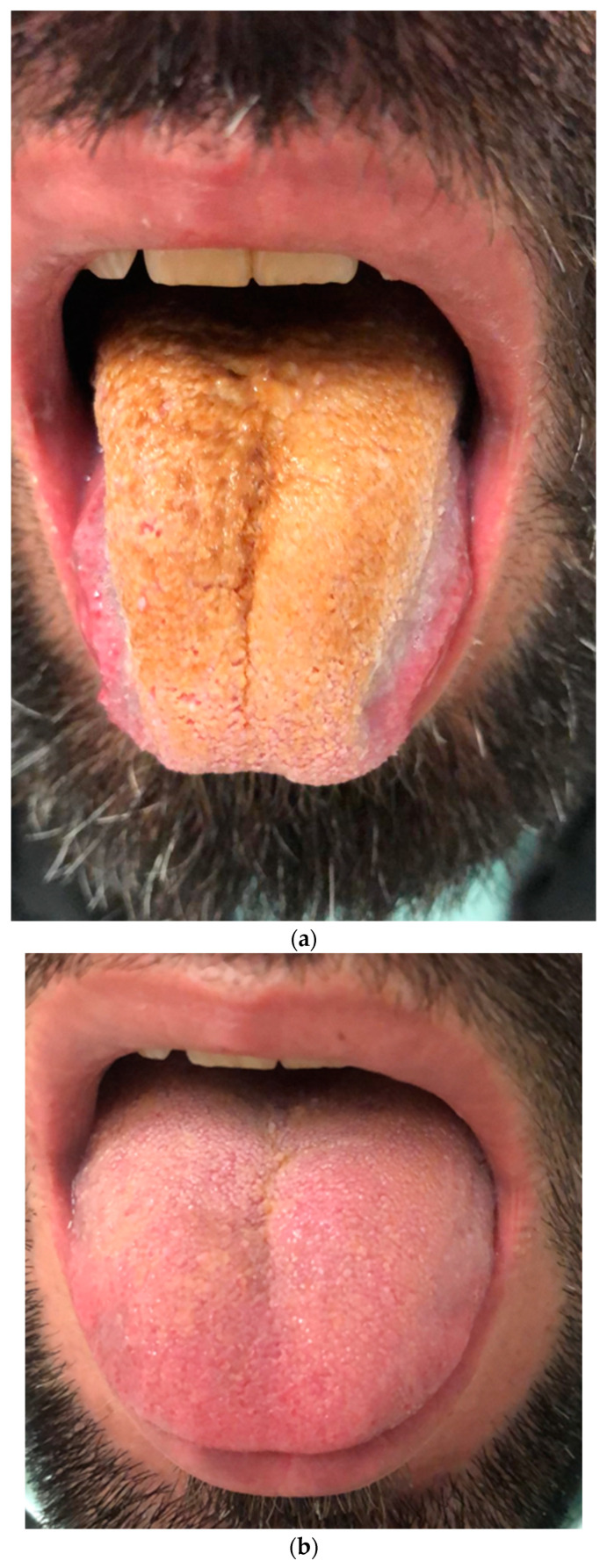
(**a**). Glossitis with fissuring in a patient with Crohn’s disease before treatment. (**b**). Improvement in glossitis with fissuring after treatment with immunomodulatory monotherapy.

**Figure 4:**
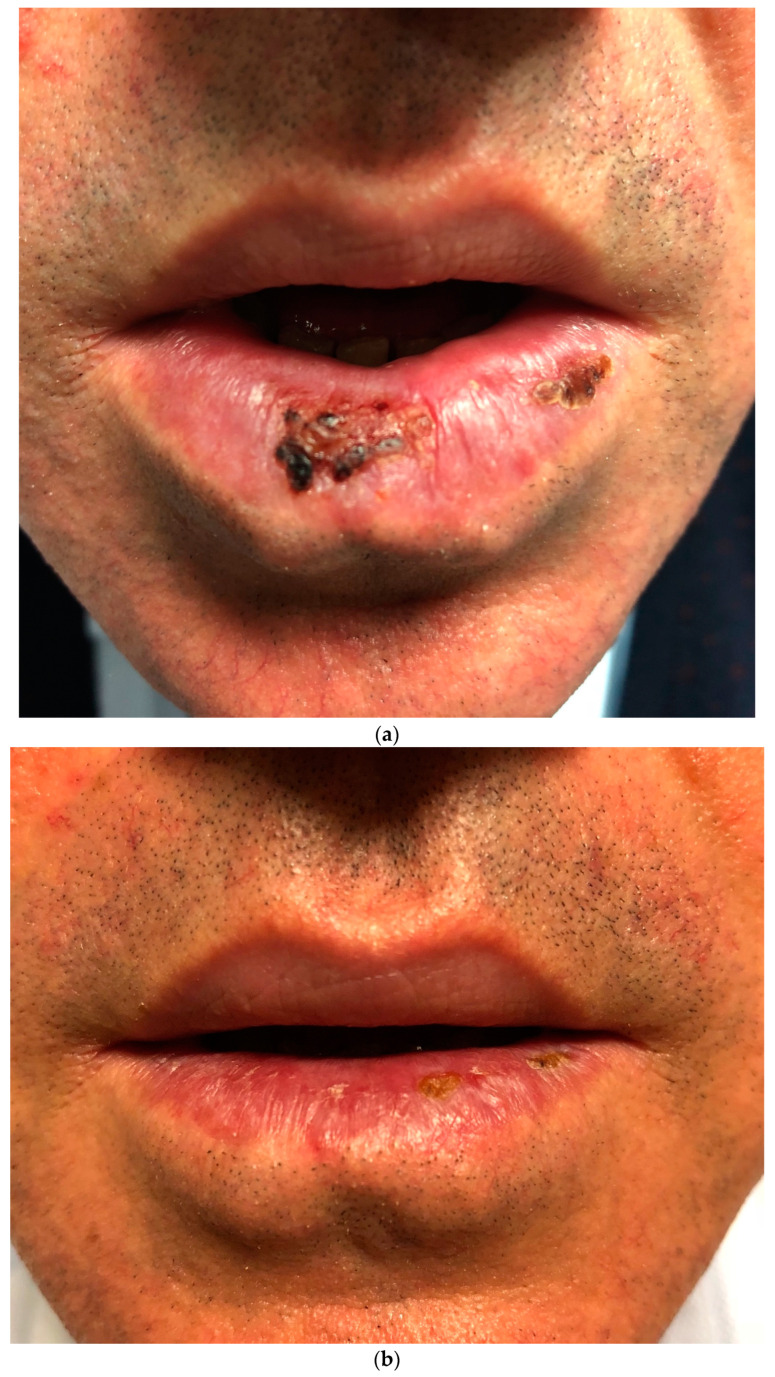
(**a**). Lip swelling with fissuring in a patient with Crohn’s disease before treatment. (**b**). Resolution of lip swelling with fissuring following treatment with a combination of immunomodulators and systemic steroids.

**Figure 5:**
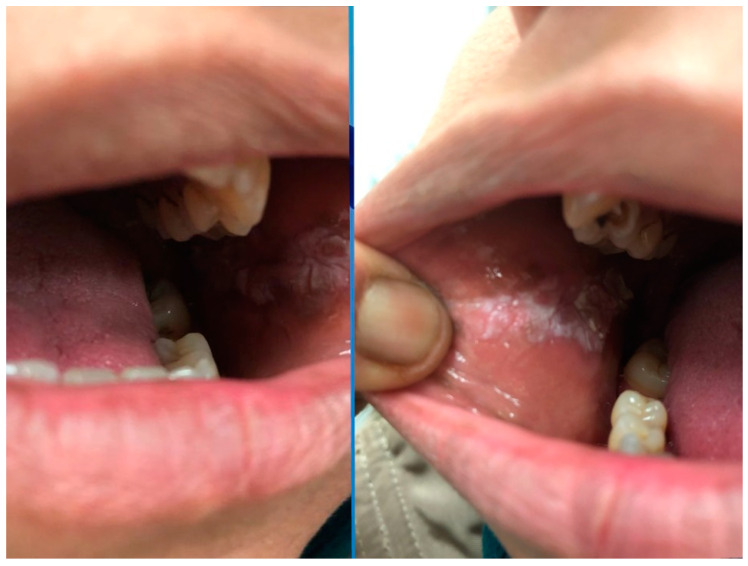
Persistent cobblestoning on the buccal mucosa in a patient with Crohn’s disease who declined biologic therapy.

Seven patients improved with IMs combined with systemic steroids or biologic therapy. The lesions regressed in all five patients who received biologic treatment. Three of them were biologic-naïve, with two patients improving with adalimumab (ADA) and one with infliximab (IFX). Two patients were already receiving biologic treatment; one was managed by switching from ADA to IFX. In this patient who underwent cobblestoning on the palatal mucosa, the oral lesion did not regress with IFX, so treatment was escalated to ustekinumab (UST), leading to lesion healing (Figure 1a; before treatment, Figure 1b; after treatment). The other patient with orofacial granulomatosis was managed by switching from ADA to UST (Figure 2). Three patients were treated with IMs with or without systemic steroids. One patient with glossitis with fissuring recovered with IM monotherapy (Figure 3a; before treatment, Figure 3b; after treatment), while another patient with lip swelling with fissuring responded to IM combined with systemic steroids (Figure 4a; before treatment, Figure 4b; after treatment). Biologic treatment was planned for the last patient when the lesion did not improve with IM and systemic steroid combination. However, the patient with cobblestoning on the buccal mucosa declined biologic therapy, and the lesion persisted (Figure 5).

## 4. Discussion

This study evaluated the treatment of specific oral lesions occurring at diagnosis or follow-up in patients with CD and its impact on clinical outcomes. Oral lesions are common in CD, with a prevalence ranging from 5% to 50% in various studies [10,18,19]. Most oral lesions are associated with active intestinal disease. Specific oral lesions, including cobblestoning, orofacial granulomatosis, lip/cheek swelling with fissuring, and pyostomatitis vegetans, are rare. The diagnosis and management of specific oral lesions can be challenging for clinicians. Multidisciplinary integrated management plans in IBD practices can improve patient outcomes and facilitate the diagnosis of specific oral lesions [11,20]. Several studies have investigated specific oral lesions in CD, with dentists participating in multidisciplinary teams [21,22,23]. Harty et al. [24] evaluated the ability of gastroenterologists to detect and accurately identify specific oral lesions at the time of diagnosis in children with CD. Consultant gastroenterologists found abnormalities in the mouth in only nine (45%) patients with oral CD. In the current study, specific lesions were also evaluated by an experienced dentist. Galbraith and colleagues [25] detected specific oral lesions in nine patients without typical clinical findings of CD. They argued that specific oral lesions may be the only finding in CD and that the diagnosis of CD should be considered in the presence of these unexplained lesions. Similarly, Vavricka et al. [2], in their cohort study of an adult population, detected oral lesions in 27.8% of patients before CD diagnosis (median time: 5 months before CD diagnosis).

The pathological explanation of oral lesions is considered to be either the spread of intestinal inflammation or an independent inflammatory event with a genetic or environmental trigger comparable to CD [2,26]. In the current study, all patients with specific oral lesions had moderate-to-severe active disease. In contrast, some adult studies have shown that oral lesions can be seen independently of disease activity [14,24,25]. This discrepancy may be better explained by previous pediatric studies: considering that oral lesions are more common in the pediatric population than in adults and that there are difficulties in diagnosing CD in children, it is plausible that oral lesions can present unrelated to disease activity in this age group. The association of nonspecific oral lesions with disease activity is widely accepted [9,10,14,15].

In addition to the treatment of intestinal inflammation and perianal disease, topical treatments with antiseptic mouthwashes and local steroids are recommended for oral lesions. Anti-TNF agents have been reported to improve the condition. While control of intestinal disease and local treatments may be sufficient for managing nonspecific oral lesions, current treatment may need to be escalated for specific oral lesions [27,28,29]. Therapeutic options include topical and systemic steroids, immunosuppressive agents, and biologic treatments [21,30]. Philips et al. [13] reported that in a multicenter study of twenty-eight patients, orofacial granulomatosis improved in twenty-three patients, with the use of anti-TNFs in nine patients, vedolizumab in one, ustekinumab in one, and thalidomide in two. However, five cases were resistant to therapies, including anti-TNFs. In the current study, specific oral lesions improved in five patients with biologic treatment and two patients with IMs with or without systemic steroids. Among patients managed with biologic treatment, three were biologic-naïve, and lesions regressed in two patients with the use of adalimumab (ADA) and one patient with infliximab (IFX). In the two patients who were already receiving anti-TNF treatment, their lesions improved after escalating to ustekinumab (UST). Biologic agents appear to be effective in managing specific oral lesions.

In the study, peripheral arthralgia/arthritis was detected significantly more frequently in patients with oral lesions (specific or nonspecific) compared to patients without oral involvement. Joint involvement that occurs in the course of Crohn’s disease may be correlated with the activation of the disease or may present independently of disease activity. Moreover, the involvement of more than one EIM is common in Crohn’s patients in whom EIMs were detected in previous studies [3,31]. There was no statistical difference when the relationship between oral involvement and poor clinical outcomes was compared among the three groups. The findings showed that oral lesions, rather than being a poor prognostic indicator, may occur in the clinical course of existing moderate-to-severe disease.

The study had some limitations. Firstly, its retrospective design and the relatively low number of patients with specific oral lesions. Secondly, although specific oral lesions were evaluated by an experienced gastroenterologist and a dentist, the diagnosis was made clinically. Lastly, whether oral lesions should be considered EIMs is a matter of debate. It has become our mainstay in evaluating specific oral lesions as EIMs because they can cause periodontitis. The strengths of the study were its long-term follow-up period, clinical visits, and regular recordings.

## 5. Conclusions

This study showed that 14.2% of CD patients had oral lesions (specific, nonspecific), and 1.2% of CD patients had specific oral lesions. Oral lesions seen in Crohn’s disease, which gastroenterologists must consider, can significantly affect patients’ quality of life and cause pain and weight loss. A multidisciplinary approach can prevent delays in diagnosis and improve patient outcomes. Close cooperation between gastroenterologists and dentists is essential for early diagnosis and optimal management. Specific oral lesions in CD were associated with active disease and immunomodulators or biologic therapy has proven to be effective in managing specific oral lesions associated with CD.

## Figures and Tables

**Table 1:** Demographic characteristics of patients with CD.

	Total *n* = 676
Age at onset of Crohn (years)	38 (11–65)
Total disease duration (years)	6.83 (0.5–29.87)
Female/Male	272 /404
Smokers (Current/Ex/None), *n* (%)	255 (37.7)/168 (24.9)/253 (37.4)
Family history of IBD, *n* (%)	94 (13.9)
CD (Disease location), *n* (%)	
Ileal (L1)	297 (43.9)
Colonic (L2)	76 (11.2)
Ileo-colonic (L3)	299 (44.2)
Upper GI disease (L4)	4 (0.6)
CD (Disease behavior), *n* (%)	
Inflammatory disease (B1)	529 (78.3)
Stenosing (B2)	45 (6.7)
Penetrating (B3)	102 (15.1)
CD P (Perianal disease)	193 (28.6)
Specific oral manifestation, *n* (%)	8 (1.18)
Cobblestoning	2
Orofacial granulomatosis	3
Glossitis (with fissuring)	2
Lip swelling (with fissuring)	2
Extra-intestinal manifestations, *n* (%)	
Aphthous ulcer	88 (13.01)
Peripheral arthralgia	166 (24.6)
Peripheral arthritis	49 (7.2)
Ankylosing spondylitis	25 (3.7)
Erythema nodosum	17 (2.5)
Sacroiliitis	11 (1.6)
Uveitis	10 (1.5)
Prımary Sclerosing Cholangitis	8 (1.2)
Episcleritis	3 (0.4)
Pyoderma gangrenous	3 (0.4)
Medication (Conventional), *n* (%)	
Mesalazine	490 (72.5)
Sulfasalazine	61 (9)
Budesonide	80 (11.8)
Steroids	291 (43)
Thiopurine	304 (45)
Methotrexate	62 (9.2)
Biological therapy, *n* (%)	
Adalimumab	132 (19.5)
Infliximab	121 (17.9)
Vedolizumab	32 (4.7)
Ustekinumab	14 (2.1)
Sertolizumab	9 (1.3)
Resective surgery, *n* (%)	229 (33.9)
Baseline CRP (mg/L), median (IQR)	6.6 (0.29–167)
Baseline HB (mg/dL), median (IQR)	13 (7.2–18)
Baseline Albumin (g/dL), median (IQR)	4.2 (2–5.2)
Baseline CDAI (CD), median (IQR)	305.5 (214.5–395)

CD = Crohn’s disease, IBD = inflammatory bowel disease, CRP = C-reactive protein, HB = hemoglobin, CDAI = Crohn’s disease activity index. Variables are summarized by median (minimum–maximum) and frequency (%).

**Table 2:** Discussion of the characteristic features of patients with CD with no oral lesions, with nonspecific oral lesions, and with specific oral lesions.

	Patients with no Oral Lesions (*n* = 580) (1)	Patients with Nonspecific Oral Lesions (*n* = 88) (2)	Patients with Specific Oral Lesions (*n* = 8) (3)	P	P 1–2	P 1–3	P 2–3
Age at onset of Crohn (years), *n* (%)	38 (11–65)	39 (14–58)	33 (18–55)	0.552	-	-	-
Total disease duration (years), *n* (%)	6.49 (0.5–29.87)	7.72 (0.61–22.76)	10.32 (5.72–16.64)	0.100	-	-	-
Smokers (current or ex), *n* (%)	364 (62.8)	56 (63.6)	3 (37.5)	0.333	-	-	-
CD (Disease location), *n* (%)							
Ileal (L1)	255 (44)	40 (45.5)	2 (25)	0.536	-	-	-
Colonic (L2)	66 (11.4)	8 (9.1)	2 (25)	0.380	-	-	-
Ileo-colonic (L3)	255 (44)	40 (45.5)	4 (50)	0.915	-	-	-
Upper GI disease (L4)	4 (0.7)	-	-	0.717	-	-	-
CD (Disease behavior), *n* (%)							
Inflammatory disease (B1)	449 (77.4)	73 (80)	7 (87.5)	0.410	-	-	-
Stenosing (B2)	40 (6.9)	5 (5.7)	-	0.684	-	-	-
Penetrating (B3)	91 (15.7)	10 (11.4)	1 (12.5)	0.560	-	-	-
CD P (Perianal disease), *n* (%)	171 (29.5)	20 (22.7)	2 (25)	0.415	-	-	-
Extra-intestinal manifestations, *n* (%)							
Peripheral arthralgia	108 (18.6)	51 (58)	7 (87.5)	**<0.001**	**<0.001**	**<0.001**	0.141
Peripheral arthritis	30 (5.2)	16 (18.2)	3 (37.5)	**<0.001**	**<0.001**	**0.008**	0.191
Ankylosing spondylitis	21 (3.6)	4 (4.5)	-	0.781	-	-	-
Sacroiliitis	7 (1.2)	3 (3.4)	1 (12.5)	0.016	0.133	**0.006**	0.298
Erythema nodosum	12 (2.1)	5 (5.7)	-	0.118	-	-	-
Pyoderma gangrenous	2 (0.3)	1 (1.1)	-	0.571	-	-	-
Uveitis	8 (1.4)	2 (2.3)	-	0.763	-	-	-
Episcleritis	3 (0.5)	-	-	0.779	-	-	-
Primary sclerosing cholangitis	8 (1.4)	-	-	0.512	-	-	-
Baseline CRP (mg/L), median (IQR)	6.7 (0.4–167)	4.75 (0.29–150)	6.9 (2–52.9)	0.418	-	-	-
Baseline HB (mg/dL), median (IQR)	13 (8.3–17.6)	12.95 (7.2–18)	15.2 (10.6–15.4)	0.782	-	-	-
Baseline Albumin (g/dL), median (IQR)	4.2 (2–5.2)	4.1 (2.7–5.1)	4 (3.9–4.7)	0.862	-	-	-
Need for steroid, *n* (%)	250 (43.1)	35 (39.8)	6 (75)	0.156	-	-	-
Biological therapy, *n* (%)	178 (30.7)	23 (26.1)	5 (62.5)	0.097	-	-	-
Resective surgery, *n* (%)	193 (33.3)	33 (37.5)	3 (37.5)	0.720	-	-	-

Significant *p* values are in bold. CD = Crohn’s Disease, GI = Gastrointestinal, CRP = C-reactive protein, HB = Hemoglobin. (-) = No. Variables are summarized by median (minimum–maximum) and frequency (%).

**Table 3:** Characteristic features of patients with CD with specific oral lesions.

	1. Case	2. Case	3. Case	4. Case	5. Case	6. Case	7. Case	8. Case	Total, *n* (%), Median (IQR)
Age at oral lesion detected (years)	46	51	68	30	47	31	23	48	46.5 (23–68)
Female/Male	M	M	M	F	M	M	M	F	2/6 (25–75%)
Smokers (Current/Ex/None)	N	N	C	C	N	C	N	N	3/5 (37.5/62.5%)
CD (Disease location)									
Ileal (L1)						**+**	**+**		2 (25%)
Colonic (L2)		**+**						**+**	2 (25%)
Ileo-colonic (L3)	**+**		**+**	**+**	**+**				4 (50%)
Upper GI disease (L4)									
CD (Disease behavior)									
Inflammatory disease (B1)	**+**	**+**	**+**		**+**	**+**	**+**	**+**	7 (87.5%)
Stenosing (B2)									
Penetrating (B3)				**+**					1 (12.5%)
CD P (Perianal disease)		**+**	**+**						2 (25%)
Resective surgery		**+**		**+**				**+**	3 (37.5%)
Extra-intestinal manifestations									
Peripheral arthralgia	**+**	**+**	**+**	**+**	**+**	**+**		**+**	7 (87.5%)
Peripheral arthritis	**+**	**+**			**+**				3 (37.5%)
Sacroiliitis				**+**					1 (12.5%)
Medication									
Current treatment before oral lesion	IM	IFX	ADA	IM	IM	IM	IM	IM	
Oral lesion treatment	IM	UST	UST	IFX	IM + steroid	IM + ADA	IM + ADA	IM + steroid	
CRP (mg/L)	21	52.9	20	151	58.7	8.9	6	50	35.50 (6–151)
CDAI	280	346	278	480	374	252	357	227	313 (227–480)

CD = Crohn’s disease, CRP = C-reactive protein (when oral lesion detected), CDAI = Crohn’s disease activity index (when oral lesion detected). Variables are summarized by median (minimum–maximum) and frequency (%) (+ = Yes) (F = female; M = male; C = current; N = none; IM = immunmodulator).

## Data Availability

The data underlying this article will be shared on reasonable request to the corresponding author.
